# Using auditory classification images for the identification of fine acoustic cues used in speech perception

**DOI:** 10.3389/fnhum.2013.00865

**Published:** 2013-12-16

**Authors:** Léo Varnet, Kenneth Knoblauch, Fanny Meunier, Michel Hoen

**Affiliations:** ^1^Neuroscience Research Centre, Brain Dynamics and Cognition Team, INSERM U1028, CNRS UMR5292Lyon, France; ^2^Ecole Doctorale Neurosciences et Cognition, Université de Lyon, Université Lyon 1Lyon, France; ^3^Integrative Neuroscience Department, Stem Cell and Brain Research Institute, INSERM U846Bron, France; ^4^Laboratoire sur le Langage le Cerveau et la Cognition, CNRS UMR5304Lyon, France

**Keywords:** classification images, GLM, phoneme recognition, speech perception, acoustic cues, phonetics

## Abstract

An essential step in understanding the processes underlying the general mechanism of perceptual categorization is to identify which portions of a physical stimulation modulate the behavior of our perceptual system. More specifically, in the context of speech comprehension, it is still a major open challenge to understand which information is used to categorize a speech stimulus as one phoneme or another, the auditory primitives relevant for the categorical perception of speech being still unknown. Here we propose to adapt a method relying on a Generalized Linear Model with smoothness priors, already used in the visual domain for the estimation of so-called classification images, to auditory experiments. This statistical model offers a rigorous framework for dealing with non-Gaussian noise, as it is often the case in the auditory modality, and limits the amount of noise in the estimated template by enforcing smoother solutions. By applying this technique to a specific two-alternative forced choice experiment between stimuli “aba” and “ada” in noise with an adaptive SNR, we confirm that the second formantic transition is key for classifying phonemes into /b/ or /d/ in noise, and that its estimation by the auditory system is a relative measurement across spectral bands and in relation to the perceived height of the second formant in the preceding syllable. Through this example, we show how the GLM with smoothness priors approach can be applied to the identification of fine functional acoustic cues in speech perception. Finally we discuss some assumptions of the model in the specific case of speech perception.

## Introduction

A major challenge in psychophysics is to establish what exact parts of a complex physical stimulation modulate its percept by an observer and constrain his/her behavior toward that stimulus. In the specific field of speech perception, identifying the information in the acoustic signal used by our neurocognitive system is crucial in order to understand the human language faculty and how it ultimately developed in human primates (Kiggins et al., 2012). In this context, questions of speech segmentation, i.e., which acoustical cues are used to isolate word units in the continuous acoustic speech stream; or phonemic categorization, i.e., which among the auditory primitives that are encoded at the neural acoustic/phonetic interface are actually used by our perceptual system to recognize and categorize phonemes, still constitute an important open debate (see Cutler, 2012 for a review). As a consequence, today there is no universal model of speech recognition that can work directly on the acoustic stream. Models of speech recognition, even the most efficient and well developed ones, usually avoid the acoustic/phonetic step (e.g., Luce and Pisoni, 1998; Norris and McQueen, 2008) or rely on systems that are not based on realistic human behaviors (Scharenborg et al., 2005).

In this paper we propose a method and procedure allowing direct estimation of which parts of the signal are effectively used by our neurocognitive system while processing natural speech. Of course one way that was used in previous work to identify relevant acoustic cues in speech is to proceed by progressive signal reductions, i.e., eliminating certain cues from the speech signal in order to demonstrate which ones are mandatory. In the 1950's, phoneme recognition was extensively studied by Liberman and colleagues for example, using the systematic variation of a limited number of features in the time-frequency domain (usually one or two) along a continuum of synthetic speech (Liberman et al., 1952, 1954, 1957). More recent work conducted on this topic has involved artificially degraded speech, such as noise-vocoded (Xu et al., 2005), sine-wave (Loizou et al., 1999), or band-pass filtered speech (Apoux and Healy, 2009). These approaches can, however, only offer a very limited account of the problem, as it is known that the speech comprehension system shows very fast and efficient functional plasticity. Once shaped by linguistic experience, our speech perception system can rapidly modify the cues that are relevant for phonemic categorization in response to drastic signal reductions or even stronger manipulations (see for example: Shannon et al., 1995). This resistance of speech perception to drastic signal impoverishment was attributed to the redundancy of information in speech: no single acoustic feature in speech is absolutely crucial for its comprehension (Saffran and Estes, 2006). The signal reduction approach can therefore not account for the many possible acoustic dimensions used by listeners in a single categorization task, or for their evolution with listening situations. While signal reduction paradigms are appropriate to study the functional plasticity triggered in our speech perception system by signal reductions, they can hardly inform us on the way the system reacts in more natural perception situations.

An alternate way to proceed would be to develop a method allowing experimenters to directly “see” where humans listen inside natural speech signals, without having to modify them. In the following, we show how a methodological solution to this issue can be provided by new developments in the domain of so-called *classification images (CIm)*. We demonstrate how this method can now be adapted to auditory experiments and how this method can further be developed to study the identification of functional fine acoustic cues in speech perception. We will also discuss how this method could be adapted to other domains of studies both in perceptual and cognitive neuroscience.

Since Ahumada and Lovell (1971) first developed a correlational technique to estimate the frequency weighting-function of observers detecting a 500-Hz tone-in-noise, much has been done for establishing a robust theoretical framework in which to describe and analyze the set of techniques gathered under the name of CIm (see Murray (2011) for an in-depth review). The basic idea underlying the classification image approach is that, faced with any kind of perceptual decision, our neurocognitive system will sometimes generate correct perceptions and sometimes errors, which could be informative on the computational mechanisms occurring in perceptual systems. If one could have access to the physical conditions of the stimulation that favor either perceptual failure or success, then one can derive the relevant parts of any stimulation that impact the perceptual decision process. As a consequence, the tasks used to generate CIm are categorization tasks. The typical paradigm used in classification image experiments is an identification or detection experiment, in which each trial consists in the presentation of one of two possible signals and the participant is instructed to classify the stimuli between the two options (*t*_0_ or *t*_1_). In order to derive a classification image, stimuli are systematically masked by a certain amount of random background-noise. For each trial, the response given by the participant, the signal actually presented and the trial-specific configuration of the noise field are recorded. The classification image aims at showing the precise influence of the noise field on the observer's response, for a given signal.

The best known (and maybe the most intuitive) method for calculating a classification image, first used by Ahumada (1996) and termed reversed-correlation, derives from the idea of establishing the correlation map between the noise and the observer's responses. In practical terms, this is done by averaging all of the noise fields eliciting response *t*_0_ and subtracting the average of all of the noise fields eliciting response *t*_1_. The idea is that if one can determine how the presence of background-noise at each point inside the space of a stimulus interferes with the decision of the observer, one can derive a map of the perceptual cues relevant to achieve a specific categorization task. By showing which components influence the recognition performance, this method gives us insights into the observer's internal decision template for this specific task. Although it has been primarily conceived as an answer to a question raised in the auditory domain (Ahumada and Lovell, 1971; Ahumada et al., 1975), and although the method could have easily been further developed to study auditory processes, this powerful tool has been mostly exploited up to now in studies on visual psychophysics. This technique has been used to investigate a variety of visual tasks, including the ability to perceive two segments as aligned or not (i.e., Vernier acuity, Ahumada, 1996), the detection of Gaussian contrast modulation (Abbey and Eckstein, 2002), the processing of illusory contours (Gold et al., 2000), visual perceptual learning (Gold et al., 2004), and luminance (Thomas and Knoblauch, 2005) and chromatic (Bouet and Knoblauch, 2004) modulation.

In the auditory domain, the classification image is a promising approach for determining which “aspects” of the acoustic signal (formant position or dynamic, energy burst, etc.) are crucial cues for a broad variety of psychoacoustic tasks (i.e., tonal or pitch discrimination, intensity perception or streaming, etc.) and particularly in the context of speech comprehension. However, to our knowledge, attempts to adapt this methodology to the auditory modality have until now produced limited results. Among noteworthy attempts, Ardoint et al. (2007) have adapted the reversed correlational method to study the perception of amplitude modulations and very similar correlational procedures have been used to determine spectral weighting functions of speech stimuli (see for example: Doherty and Turner (1996); Apoux and Bacon (2004) or Calandruccio and Doherty (2008)). Two severe limitations can, at least partly, explain the limited development of the technique. Firstly, several thousands of trials are typically needed to compute a classification image accurately. The minimum number of trials theoretically required is equal to the number of free-parameters, but many more are needed to be able to estimate the classification image with an adequate signal-to-noise ratio (up to 11400 trials, in Barth et al., 1999). This problem has been overcome in the visual domain by reducing the number of random variables under consideration, for example by averaging along irrelevant dimensions (Abbey and Eckstein, 2002, 2006), or by using a “dimensional” noise (Li et al., 2006). Unfortunately, none of these methods can be used with such complex and time-varying stimulus as speech. Furthermore, mental and physiological fatigue occurs rapidly when listening to very noisy stimuli. The second factor restricting the use of reverse-correlation for estimating auditory CIm is the strong assumption about the statistical distribution of the noise imposed by the statistical theory. Since its theoretical background has mostly been developed assuming additive Gaussian-noise, methods such as reverse-correlation are not the most suitable statistical framework to deal with non-Gaussian noise-fields. In the visual domain, CIm can be based on Gaussian noise, for example in the case of luminance noise which will modify the observer's perception in a symmetric fashion, adding or subtracting luminance to pixels in a picture. The interest of using CIm for the study of speech signals, however, imposes the use of acoustic stimuli which will have complex spectro-temporal composition and the calculation of an auditory classification image should therefore not be based on the amplitude of the noise samples, but rather on the power of the time-frequency bins of their power spectrum. These unfortunately generally have non-Gaussian distributions. These two limitations make it difficult to calculate auditory CIm using the standard reverse-correlation method.

A major advance in the comprehension and computation of CIm was achieved by Knoblauch and Maloney (2008) who proposed to fit the data with a Generalized Linear Model (GLM), which provide a more accurate and comprehensive statistical framework for calculating CIm. This initial work was followed by Mineault et al. (2009) and Murray (2011), (2012). Interestingly, this appealing approach offers a way to overcome the two pitfalls mentioned above. Firstly, GLMs naturally allow the addition of prior knowledge on the smoothness of the expected classification image, resulting in Generalized Linear Models (GLMs) (Hastie and Tibshirani, 1990; Wood, 2006). By exploiting the dependencies between adjacent noise values, one can significantly reduce the number of trials required. In fact, GLMs with priors are widely used for describing the stimulus-response properties of single neurons (Pillow, 2007; Pillow et al., 2008), in particular in the auditory system (in terms of Spectrotemporal Receptive Field, STRF, Calabrese et al., 2011). Secondly, unlike the reverse-correlation method, the GLM does not require the noise to be normally distributed. Accordingly, it can measure CIm using noise fields from non-Gaussian distributions, such as the power spectrum of an acoustic noise, in a similar way to the calculations of second-order CIm using GLM by Knoblauch and Maloney (2012). Therefore, Generalized Linear and Additive Models provide suitable and powerful tools to investigate the way in which the human system achieves fast and efficient categorization of phonemes in noise. In this paper we applied the GLM with smoothness priors technique to the identification of acoustic cues used in an identification task involving two VCV speech sequences: ABA (/aba/) and ADA (/ada/). In this particular case a strong hypothesis, formulated in Liberman et al. (1954), is that the second formant transition would be a key for classifying the stimulus into [ABA] or [ADA]. Under this assumption, the classification image would be focused on the time-frequency localization of the second formant transition.

## Materials and methods

In the following sections we use the convention of underlined symbols to indicate vectors, double underlined symbols to indicate matrices, and non-underlined symbols to indicate scalars.

### Experimental procedure

Three native French-speaking listeners took part into this study: the first two Léo Varnet and Michel Hoen are co-authors on the paper and were not naïve regarding details of the study, a third participant was thus added, S.B. who was completely naïve toward the task. They were 24, 25, and 35 year old, males, right handed and native French speakers, without known language or hearing deficits.

Targets sounds, hereafter denoted *t*_0_ and *t*_1_, were two natural-speech signals (ABA /aba/ and ADA /ada/ respectively) obtained by concatenating the same utterance of /a/ with an utterance of /ba/ or /da/. Original sounds were recorded in a soundproof chamber by the same female speaker and digitized at a sample rate of 44.1 kHz. The sound samples were 680 ms long, and their average power was normalized. Each stimulus *s*_*i*_ consisted of one target-sound, embedded in a Gaussian additive-noise using Equation (1):

(1) $${\underline{s}}_{i}=\alpha_{i}\cdot {\underline{t}}_{k_{i}}+{\underline{n}}_{i}$$

In (1), *i* is the trial number, *k_i_* the target number (0 or 1) associated with this trial, *n*_*i*_ the noise field drawn from a normal distribution, and α_*i*_ a factor allowing the adjustment of signal-to-noise ratio (SNR) as a function of the participant's behavior, see Adaptive stimulus-delivery procedure below. Each stimulus was normalized in intensity level using the root mean square and preceded with a Gaussian fade-in of 75 ms convolved with a Gaussian-noise, in order to avoid clicks or abrupt attacks. The sample rate was the same as for the original sounds.

The experiment consists in the presentation of a list of *N* = 10,000 noisy stimuli (5000 for each target) presented in a completely random order. Participants were instructed to listen carefully to the stimulus and then indicate by a button press whether the masked signal was *t*_0_ or *t*_1_, a response denoted by *r*_*i*_ (= 0 or 1). The following trial began after 200 ms. Listeners could complete the task over a period of 1 week, at their own pace, depending on individual fatigue and availability, for a total duration of approximately 3h. Each participant divided the experiment in sessions of approximately 1000 stimuli, on their own initiative. The experiment was run in a quiet experimental room and stimuli were delivered using Sennheiser's HD 448 headphones.

### Adaptive stimulus-delivery procedure

During the course of the experiment, the signal level was constantly adjusted to ensure a correct response rate around 75%, as in several previous classification image experiments (e.g., Gold et al., 2000). Signal contrast must be strong enough to ensure that the SNR will not severely affect the decision rule, but sufficiently low so that noise influences the decision of the observer. That is to say, that noise must be misleading on a sufficient number of stimuli, without leading the observer to reply randomly on the task. For this purpose, the SNR was varied from trial-to-trial on the basis of a local rate of correct responses calculated on a 10-trial window, with an adaptation of 0.2, 0.4, 0.6 or 0.8 dB for differences of 5, 10, 15, or 20% between intended and actual scores (variations of the SNR were limited to the range −20 dB to −0 dB; we systematically record the final SNR value for one session and use it as starting value for the next session before the adaptive algorithm takes over in adjusting the SNR). However, in the following we assume that the SNR does not affect the observer's strategy for categorization, a point that will be discussed in Discussion.

### Deriving auditory classification images

**Figure 1:**
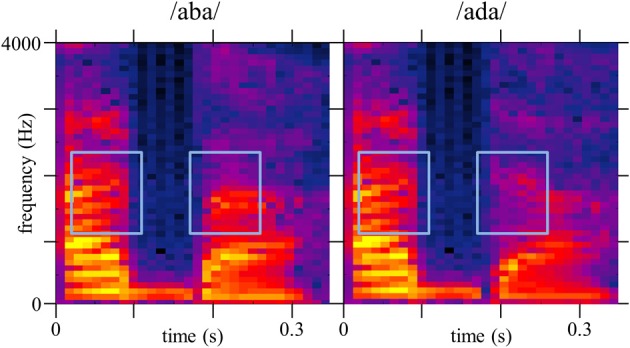
**Spectrograms of target-signals *t*_0_ (/aba/) and *t*_1_ (/ada/) used for the vectorized spectrograms *T*0 and *T*1, on a logarithmic scale (dB)**. Blue boxes indicate the second formantic transition (F2).

Each stimulus noise *n*_*i*_ is characterized by its power spectrogram, whose components are entered as predictor variables in the model. Power spectrograms were calculated with Matlab function *spectrogram*, using a Short-Time Fourier Transform with a moving 512 points Hamming window and no overlap, resulting in 86.13 Hz frequency resolution and 11.6 ms temporal resolution. Since the last 340 ms of the signal were almost silent, we limited our analysis to a time range of 0–0.34 s and a frequency range of 0–4048 Hz, thus ensuring that the size of the data-set would not exceed computational limits. The resulting 46-by-30 matrix (frequency bins by time bins) is reshaped into a 1380-by-1 vector of time-frequency bins, labeled *X*_*i*_. A similar treatment is applied to both targets, resulting in vectorized power spectrograms *T*_0_ and *T*_1_ (Figure 1).

More biologically-inspired time-frequency representations of the noise, as a cochleagram, can replace the spectrogram for deriving the CIm, in order to obtain a “higher-level” representation of the functional acoustic cues. Or more simply, we could apply a logarithmic scale to the frequency axis to account for the non-linear spacing of filter center frequencies, as it is done in the STRF calculation. Nevertheless, in our case the aim was only to replicate a known property of the speech comprehension system, which is more intuitive on a simple time-frequency representation.

In general agreement with the literature on classification image (for example Ahumada, 2002) we assume that the observer performs the detection of acoustic cues linearly by template matching, a longstanding model for decision making. First, an internal decision variable *d*_*i*_ is computed by taking the scalar product of the input with a weighting function *w* referred to as the observer's template, and adding a random variable ε*i* representing the internal noise of the system (accounting for the fact that the observer does not necessarily give the same response when presented with the same stimulus twice). In (2), the errors ε*i* are assumed to have a zero mean symmetric distribution and to be independent from trial to trial.

(2) $$d_{i}={\left({\underline{X}}_{i}+{\underline{T}}_{k_{i}}\right)}^{T}\cdot \underline{w}+\varepsilon_{i}$$

Then the response variable is given by (3):

(3) $$r_{i}=\left\{ \begin{array}{l} \;1\text{ if }{\text{d}}_{i}>c\\ 0\text{ otherwise} \end{array}\right.$$

*c* is a fixed criterion that determines the bias of the observer toward one alternative. Knoblauch and Maloney (2008) reformulated this very simple model in terms of a GLM, by expressing the probability that the observer gave the response *r*_*i*_=1, given the data *X*_*i*_, in the case of presentation of the target number *k*_*i*_:

(4) $$P(r_{i}=1)=\Phi ({\underline{X}}_{i}{}^{T}\cdot \underline{\beta }+{\text{s}}_{k_{i}})$$

with Φ cumulative distribution function associated with ε, β the classification Image, and *s* a two-level factor reflecting the influence of the target actually presented on the response. In line with the psychophysics literature, we could assume that ε is taken from a logistic distribution (a common choice for modeling binomial data), and therefore the associated psychometric function Φ will be the inverse of the logit function. It would still be possible to use other assumptions, as a Gaussian distribution for ε and as a consequence, the inverse of the probit function as Φ. Such changes might have an impact on the model though it would be presumably small.

The structure of Equation (4), with a linear combination of parameters linked to the dependent variable via a psychometric function, is the typical form of a GLM (Fox, 2008; Knoblauch and Maloney, 2012). At this stage we could thus determine the values of the model parameters θ = {β, s} that best fit the empirical data, by simply maximizing the log-likelihood:

(5) $$\begin{array}{l} L\left(\underline{\theta }\right)=\log\left(P\left(\underline{r}|\underline{\theta },\underline{k},\underline{\underline{X}}\right)\right)\\ \;=\;\log\left(\prod_{i}P\left(r_{i}|\underline{\theta },k_{i},{\underline{X}}_{i}\right)\right) \end{array}$$

that is a natural measure of match between data and fit, assuming statistical independence between responses *r*_*i*_. Thus, calculating:

(6) $${\underline{\hat{\theta }}}_{ML}=\underset{\underline{\theta }}{\text{argmax}}L\left(\underline{\theta }\right)$$

by a standard maximization algorithm (e.g., the built-in Matlab function *glmfit*) would provide us maximum likelihood estimates of the CIm, $\hat{\beta }$ and of the stimulus factor *s*.

Unfortunately, these estimates would be presumably too noisy to be decipherable. Indeed GLMs, as well as reverse-correlation, when comprising a large number of predictors (1382 in this example), are prone to overfitting, which means that the model will describe the trial-dependent noise as well as the underlying classification mechanism. Estimates of the observer's template by GLM can therefore be quite noisy, and the model will not be able to generalize to novel data. For proper predictions of previously unseen data, templates should not be determined by the specific distribution of noise in trials used to fit the model, but rather reflect the decision process of the observer.

One solution has been developed in the GLM framework under the name “Penalized Likelihood,” which has been widely used for estimating the receptive fields of single neurons (Wu et al., 2006; Calabrese et al., 2011; Park and Pillow, 2011) and adapted to CIm by Knoblauch and Maloney (2008) and later by Mineault et al. (2009). Another example of using a similar method for an application in the auditory domain can be found in Schönfelder and Wichmann (2012), who modeled results from a classical auditory tone-in-noise detection task using this approach. Among the various R or Matlab toolboxes available, we decided to use Mineault's function glmfitqp (http://www.mathworks.com/matlabcentral/fileexchange/31661-fit-glm-with-quadratic-penalty) that allows optimizing a GLM with quadratic penalty. The aim of this method is to incorporate prior knowledge about the smoothness of the intended classification image (which is equivalent to introduce dependencies between adjacent predictors). To do so, we associate with each value of the model parameters θ a probability *P*(θ|λ) representing our a priori beliefs about the true underlying template (in our case, a smoother classification image will be more expected, and therefore have a high prior probability). This prior is defined by a distribution and a set of hyperparameters λ, as explained later. Then, instead of maximizing the likelihood as before, we maximize the log of the posterior *P*(θ | *r*, *k*, *X*, λ) that takes into account the likelihood and prior information, as evidenced with Bayes' rule:

(7) $$P\left(\underline{\theta }|\underline{r},\underline{k},\underline{\underline{X}},\underline{\lambda }\right)=\frac{P\left(\underline{r}|\underline{\theta },\underline{k},\underline{\underline{X}}\right)\cdot P\left(\underline{\theta }|\underline{\lambda }\right)}{P(\underline{r}|\underline{k},\underline{\underline{X}})}$$

Therefore the Maximum A Posteriori (MAP) estimate of the model parameters is given by:

(8) $$\begin{array}{l} {\hat{\underline{\theta }}}_{\text{MAP}}={\text{argmax}}_{\underline{\theta }}\log\left(P\left(\underline{\theta }|\underline{r},\underline{k},\underline{\underline{X}},\underline{\lambda }\right)\right)\\ \;=\underset{\underline{\theta }}{\text{argmax}}\log\left(P\left(\underline{r}|\underline{\theta },\underline{k},\underline{\underline{X}}\right)\cdot P\left(\underline{\theta }|\underline{\lambda }\right)\right)\\ \;=\underset{\underline{\theta }}{\text{argmax}}\left[\log\left(P\left(\underline{r}|\underline{\theta },\underline{k},\underline{\underline{X}}\right)\right)+\log\left(P\left(\underline{\theta }|\underline{\lambda }\right)\right)\right]\\ \;=\underset{\underline{\theta }}{\text{argmax}}\left[L\left(\underline{\theta }\right)+R\left(\underline{\theta }\right)\right] \end{array}$$

The last equation can be seen as the same maximization of the log-likelihood as before, with an additional penalty term, *R*(θ), that biases our estimate toward model parameters with higher a-priori probability. The optimal estimate is a tradeoff between fitting the data well and satisfying the constraints of the penalty term. Therefore, a prior on smoothness will favor CIm with slow variations in time and frequency (but note that other types of priors exist (Wu et al., 2006), e.g., implying assumptions on independence (Machens et al., 2004), sparseness (Mineault et al., 2009; Schönfelder and Wichmann, 2012), or locality (Park and Pillow, 2011) of the model parameters).

In agreement with the Matlab function we use, we chose our smoothness prior to be a sum of two quadratic forms:

(9) $$P\left(\underline{\theta }|\lambda_{1},\lambda_{2}\right)=\lambda_{1}{\underline{\theta }}^{T}{\underline{\underline{L}}}_{1}\underline{\theta }\;+\;\lambda_{2}{\underline{\theta }}^{T}{\underline{\underline{L}}}_{2}\underline{\theta }$$

where *L*_1_ is the Laplacian matrix along dimension 1 (time), *L*_2_ the Laplacian matrix along dimension 2 (frequency) in the time-frequency space (Wu et al., 2006). Thus, the quadratic form θ^*T*^ *L*_*D*_ θ provides a measure of the smoothness of θ over dimension *D*. As we do not know the appropriate importance of smoothness along the time and frequency axis, we introduce two hyperparameters (indeed the scale of smoothness in the spectral and temporal dimensions are presumably unrelated) λ = λ_1_, λ_2_ that control the prior distribution on θ, and therefore the strength of penalization. The absolute values of the hyperparameters (also called “regularization parameters”) have no clear interpretation, as they represent the relative importance of quality of fit and smoothness. For large (>1) values of λ_1_ and λ_2_ we put a strong disadvantage on sharp CIm, and for λ_1_= λ_2_ = 0 we recover the initial maximum likelihood solution.

The standard method for setting the value of the hyperparameters is cross-validation (e.g., in Wu et al., 2006; Schönfelder and Wichmann, 2012). This approach involves a partition of the data between a “training” and a “test” set. For each given couple of hyperparameters, we can estimate the model parameters on the training-set by maximum a posteriori, as explained previously. It thus becomes possible to assess how the model parameters would generalize to an independent dataset, by comparing the predicted responses on the test-set to the actual responses. When the model predicts the most accurately unseen data, the strength of priors is considered as optimal.

We determined one single couple of optimal hyperparameters {λ_1, opt_, λ_2, opt_} by participant. More precisely, the selection of λ_1, opt_ and λ_2, opt_ is performed on a model gathering together trials on which signal 0 or 1 was presented, following the equation:

(10) $$P\left(r_{i}=1\right)=\Phi ({\underline{X}}_{i}{}^{T}\cdot \underline{\beta }+b)$$

This GLM relates strongly to that derived from Equation (4), except that it does not take into account information about the target signals that was actually presented at each trial (the two level factor *s* being replaced with a constant term *b*). In particular this simple linear model cannot account for the fact that when presented with a masked target *t*_*i*_ the observer is more likely to respond *r*_*i*_ and as a consequence, it yields less accurate predictions. Nevertheless, because the estimated template $\hat{\beta }$ is very close to that derived from Equation (4), this model provides a good basis for estimating a common set of optimal hyperparameters, which will then be applied in all estimations of CIm for this subject. To do so, we plotted the 10-fold cross-validation rate of the model as a function of the hyperparameters {λ_1_, λ_2_} used for fitting this model. The optimal hyperparameters {λ_1, opt_, λ_2, opt_} are found by choosing the models that yielded the best prediction of responses to a new data set i.e., that correspond to a maximum of cross-validation rate. When the function exhibits several peaks, the values are chosen to favor smooth weights along the two dimensions. The same procedure was repeated for both participants. In more simple terms, this technique yields to a form of Automatic Smoothness Determination (Sahani and Linden, 2003) allowing us to apply smoothing in a principled fashion.

We assessed the statistical significance of the weights in the resulting CIm by a simple permutation test. “Resampled” estimates of the CIm were computed from 500 random re-assignment of the responses to the trials (i.e., random permutation of the response vector *r*). We therefore obtained estimates of the distribution of weights at each time-frequency bin, under the null hypothesis of no effect of noise at this time-frequency bin. We used these estimated distributions to highlight weights significantly different from 0 (*p* < 0.005, two-tailed) in the actual CIm.

## Results

**Figure 2:**
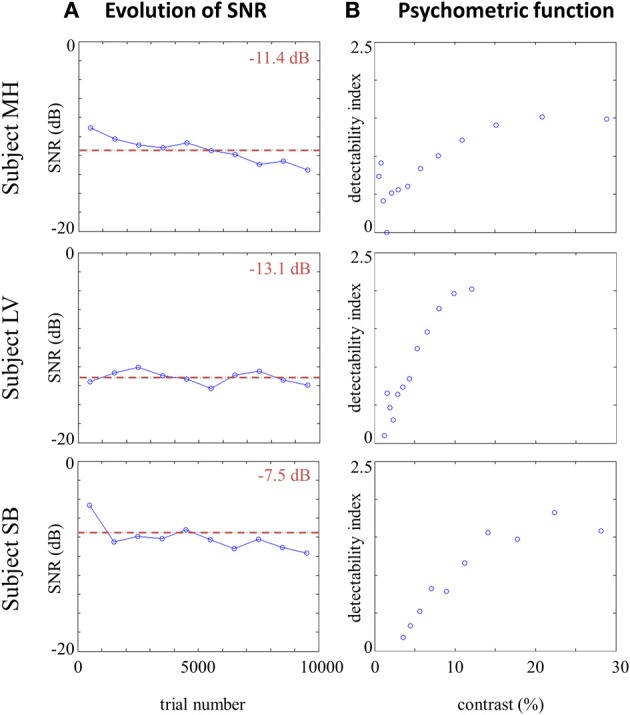
**(A)** Evolution of SNR across trials (mean SNR by blocks of 1000 trials) for each participant, and overall mean SNR (red dotted line). **(B)** Psychometric function of each participant: detectability index *d*' (defined as *d*' = Φ^-1^ (*P_H_*)- Φ^-1^(*P_FA_*) as a function of signal contrast (values calculated on less than 20 observations are not included).

The SNR was manipulated across trials via an adaptive procedure, in order to maintain the percentage of correct answers roughly equal to 75% during the course of the entire experiment. In consequence, variations of SNR provide an overview of observers' performances in the phoneme categorization task. Figure 2A plots the evolution of SNR during the experiment and the mean SNR for each participant, showing that there is no strong effect of perceptual learning as a decline of SNR for the same performance level over the course of the experiment. The psychometric functions are therefore estimated on all available data for each participant (Figure 2B). As noted by Eckstein et al. (1997), linear observers such as the one described in Equations (2) and (3) produce a linear relationship between signal contrast and detectability index (defined here as *d*' = Φ^−1^(*P_H_*) − Φ^−1^(*P_FA_*) with *P_H_* the proportion of response 1 when signal *t*_1_ was presented and *P*_*FA*_ the proportion of response 1 when signal *t*_0_ was presented). For the real data, such a linear relationship can be observed in the range 2–10% signal contrast, supporting our assumption of a (at least locally) linear model for the observers. Furthermore, the small number of trials corresponding to very high or very low contrast could also account for the non-linearity.

**Figure 3:**
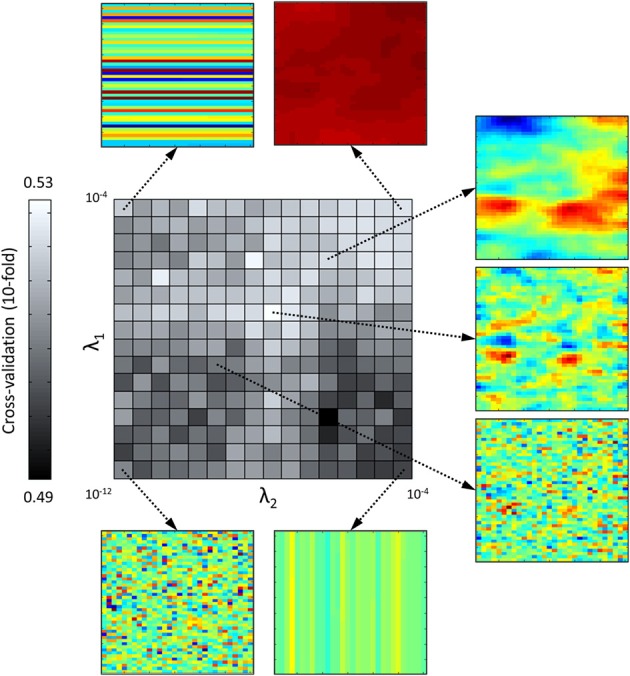
**Prediction accuracy of the model (in terms of 10-fold cross-validation rate) as a function of regularization parameters λ_1_ (x-axis) and λ_2_ (y-axis) in logarithmic scale, for one participant (MH)**. Around are shown classification images obtained with different pairs of regularization parameters (λ_1_, λ_2_) (*n* = 10000 trials for each estimate).

As explained in the Methods section, an optimal set of hyperparameters is chosen by plotting the cross-validation rate of the model derived from Equation (4) and fitted by MAP estimate as a function of {λ_1_, λ_2_}. An example of resulting surface for subject MH is shown on Figure 3, with a clear maximum at λ_1_ = 3.16e-08, λ_2_ = 1e-08 (a similar pattern of cross-validation rate is seen for the four other subjects). The low values of cross-validation rate, ranging from 0.49 to 0.53, are explained by the fact that the very simple model described in Equation (10) does not take into account information about the target signal presented, which is critical for an accurate prediction of observer's responses. Nevertheless, it allows us to track how the calculated template generalizes to new data sets, excluding predictors other than noise. For low values of hyperparameters, the model is overfitted and cannot generalize to the “test” dataset, resulting in prediction performances around chance level (50%). For high values of hyperparameters, the classification image is flat and the model always gives the same answer, which corresponds to the response bias of the observer (in this case 52% of Michel Hoen's answers were “aba”). In between a couple of hyperparameters may be found that maximizes prediction performances.

**Figure 4:**
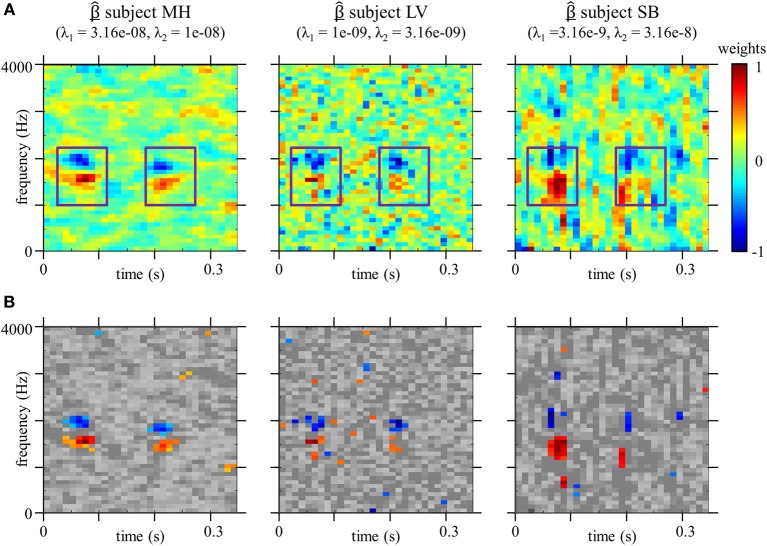
**(A)** Classification Image $\hat{\beta }$ for each participants, estimated with optimal smoothness hyperparameters λ_1_ and λ_2_ (*n* = 10000 trials for each estimate). Weights are divided by their maximum absolute values. Boxes corresponds to the position of the second formantic transition (F2) in the original stimuli spectrograms. **(B)** Same as above except that non-significant weights are shown in gray scale (*p* < 0.005, permutation test).

Figure 4A shows the CIm $\hat{\beta }$ obtained by the GLM method with smoothness priors, as well as the optimal values of λ_1_ and λ_2_ for the three listeners. For each participants, the classification image provides a measure of the strength of the relation between the noise at different time-frequency locations and the speech identification scores. In that sense, the classification image may be regarded as a measure of the contribution of each time-frequency bin to categorization, with high absolute values for locations at which the power of the noise influences the decision of the observer. As can be seen from Figure 4, CIm often exhibit both positive (red) and negative (blue) weights corresponding to areas where the presence of noise, respectively, increases or decreases the probability of stimulus to be identified as signal *t*_0_ (/aba/) (weights are divided by their maximum absolute value to provide a common basis for comparison). Figure 4B shows the same classification image, but non-significant weights are represented in gray tones (*p* < 0.005, permutation test), as explained in the method section.

**Figure 5:**
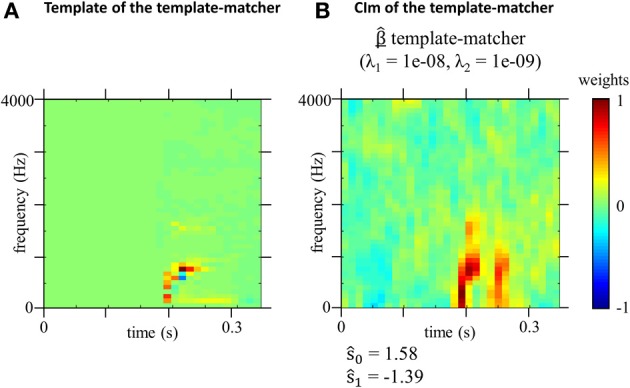
**(A)** Difference template *w* used by the template matcher (difference between spectrograms of the targets). **(B)** Estimated model parameters for the template-matcher optimal hyperparameters λ_1_ and λ_2_ (*n* = 10000 trials). Weights are divided by their maximum absolute values.

For a better understanding of these CIm, we ran a similar test performed by an ideal template-matcher (Figure 5). This classifier is the optimal observer for the linear model presented in Equation (2) and (3) and is defined by taking *w* =(*T*_1_ − *T*_0_)/*K* with *K* a normalization constant (difference template shown on Figure 5A), and *c* = 0. Note that as it is used by the template-matcher as a linear weighting function, we represented it with a linear scale, whereas speech spectrograms on Figure 1 are classically represented using a logarithmic scale (dB). Since the difference template corresponds to the difference between the two target spectrograms, the template-matcher observer bases its classification strategy on the time-frequency bins where the spectrograms of the two signals differ most in terms of power (corresponding in this case to the region of the onset of the first formant, which appears in red on the difference template). As the performances of the algorithm do not vary over time, the SNR for stimulus presentation was set to −25 dB, for a resulting percentage of correct answers of 68%. The difference template and the obtained CIm are plotted on Figure 5.

The classification image obtained from the optimal observer (Figure 5) is very different that those obtained from human listeners (Figure 4), suggesting that the usage of acoustic cues by the human speech perception system is suboptimal in this particular example.

## Discussion

By providing insight into how a given noise distribution affects speech identification, the GLM may help to better understand the perceptual mechanisms behind speech-noise interferences. With the present study, we demonstrated that CIm obtained from the categorization of natural speech signals, i.e., the phonemes /b/ and /d/ embedded in /aba/ and /ada/ logatomes, can offer insight into the way in which the human speech perception system achieves fast and efficient categorization of phonemes in noise. By adapting the GLM with smoothness priors to an adaptive identification task performed on speech stimuli, we have shown that CIm are applicable to studies in the auditory modality and can be used to identify relevant portions of speech.

### Auditory classification images from a phoneme categorization task

Because the optimal values obtained for the smoothing parameters λ_1_and λ_2_ are not the same for all participants, the calculation yields smoother CIm for MH than LV, and SB (left vs. middle and right panel on Figure 4). Nevertheless, a similar pattern of weights is observed for both participants. If we map the CIm obtained from our human listeners onto the original stimuli spectrograms (Figure 1), we can observe two main foci of high- and low-value weights, located in the time-frequency domain exactly over the second formant F2 (blue frames in Figure 4). More precisely, our preliminary observations suggest that, unlike the template-matcher, which bases its decisions on main energy differences between the two signals, the human observers used for categorizing /aba/ and /ada/ speech specific cues, namely the end of F2 in the first vowel and the onset of F2, on the consonant, just following the occlusion. This is in agreement with previous findings by Liberman et al. (1954): they showed that the second formantic transition can serve as a cue for classifying phonemes into /b/ or /d/, by using synthetic speech and by modulating the direction and extent of the second formantic transition. However, they did not test all possible cues and limited their study to manipulations of F2, leaving open the possibility that other portions of the signal could also be identified as functional cues for this categorization task. Our approach conversely takes into account all possible acoustic cues which might be used in the categorization and thus the results provides stronger support for the hypothesis that the second formantic transition is the only crucial characteristic for performing the task.

The pattern consistently observed at each time-frequency locations of the second formantic transition, composed of a cluster of positive weights below a cluster of negative weights, supports the assumption that frequency information is coded in terms of relative difference across frequency bands (Loizou et al., 1999). When the energy of the noise is concentrated around 2000 Hz during the formantic transition, the second formant sounds higher than it actually is, and therefore the consonant is more likely to be perceived as /d/. On the contrary, a high noise power around 1500 Hz biases the decision toward /b/. In both cases, this phenomenon is strengthened by a similar distribution of the noise at the end of F2 in the preceding vowel /a/. Indeed, the strong absolute values of weights around 0.075 s for frequencies between 1500 Hz and 2200 Hz in Figure 4, indicates that the decision depends on this region, even though it contains no useful information for performing the task (in our experiment the first syllable was the same for both stimuli because it was obtained by concatenating the same utterance of /a/). A very similar pattern of weights has been observed for a Vernier acuity task in the visual domain (Ahumada, 1996), highlighting the fact that our phoneme categorization task could be seen as the detection of the alignment of formants in time. In addition, the obtained CIm evidence the fact that the estimation of the second formant by the auditory system is a relative measurement, since the presence of noise masking the position of the second formant in the preceding vowel influences the decision of the observer. This is in-line with theories postulating phonemic perception as an interpretation of phonetic movements and trajectories. Further work will be dedicated to studying in details the relationship between classification image and phonetic discriminations.

This simple example illustrates the fact that our method is suitable for studying the processing of fine-acoustic cues during speech categorization by the human speech perception system. Indeed, the use of a GLM with smoothness priors as a statistical method for the estimation of CIm in the auditory modality is a reasonable way of overcoming traditional limitations of this methodological approach in the auditory modality.

**Figure 6:**
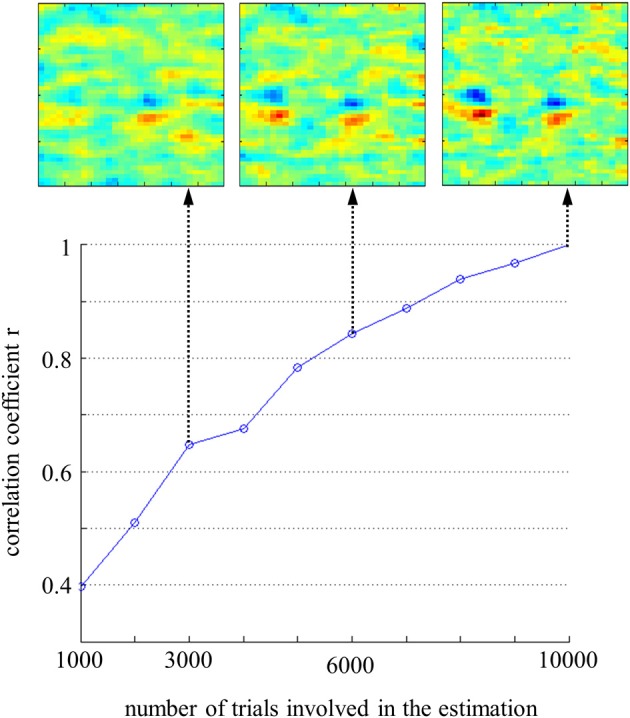
**Correlation between coefficients of the Classification Images estimated on *n* trials and the “overall” Classification Image, for participant MH**. Examples of Classification Images are shown at 3000, 6000, and 10,000 trials.

First, this method allows the addition of prior knowledge about the smoothness of the expected image. By exploiting the dependencies between adjacent noise values, one can significantly reduce the number of trials required to obtain a reliable classification image. Since our goal here was to explore the possibilities of the method, participants completed a very large set 10,000 trials, in order to gather sufficient amount of information and data to be able to run accurate simulations. Nevertheless, there is in fact no need for so many trials to calculate a classification image. To get an idea of the appropriate amount of data, we estimated the model parameters at various stage of completion of the experiment for participant Michel Hoen, and calculated their correlation with the “overall” CIm (calculated on 10,000 trials) as a measure of accuracy (Figure 6). It can be seen from this graphs that we reached the level of *r* = 0.8% with approximately 6000 trials, and therefore this amount of data can be considered as sufficient to calculate a reliable estimate of the underlying template. On the other hand, below 6000 trials the optimal set of hyperparameters becomes very difficult to identify because the cross-validation rate exhibits several peaks and a less typical profile.

Second, unlike the reverse-correlation method, the GLM does not require the stimulus or the noise to be normally distributed. Accordingly, it can efficiently measure CIm using noise-fields with non-Gaussian distributions, such as the power spectrum of an acoustic-noise, in a similar way to the calculations of second-order CIm using GLMs (Barth et al., 1999; Knoblauch and Maloney, 2012). It should be noted that we could also rely on the Central Limit Theorem and assume that images are normally distributed, as long as the noise is not heavy-tailed, but this approximation leads to less precise estimation and to far less smooth CIm. In this experiment we used white noise in order to mask equally acoustic cues at low and high frequencies; however, it is known that the human auditory system does not perceive all frequency octaves with equal sensitivity (Robinson and Dadson, 1956; Suzuki and Takeshima, 2004). One option could be to use another spectral distribution that compensates for the weighting function of the auditory system, like pink noise in which all frequency octaves are assumed to be perceived with an equal loudness.

As mentioned earlier, our approach based on GLM with smoothness priors has the advantage that it does not make any assumptions about the distribution of noise in the stimulus, unlike the reverse correlation approach. Nevertheless, other strong assumptions about how observers perform speech categorization tasks in noise have been made or maintained and must be discussed.

### Spectrotemporal alignment of targets

**Figure 7:**
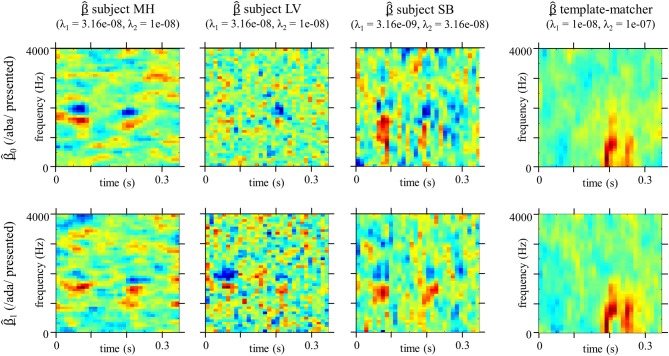
**Classification Images $\hat{\beta }$_0_ and $\hat{\beta }$_1_ estimated on the trials where *t*_0_ (/aba/) or *t*_1_ (/ada/) was presented respectively (*n* = 5000 trials for each estimate)**. Hyperparameters values are the same as for the “overall” Classification Images Figure 4. Weights are divided by their maximum absolute values.

The first simple requirement for observing functional cues involved in our identification task is the precise spectrotemporal alignment of the two targets. As we want to know in which time-frequency bins the listener is focusing, the acoustic cues of interest must be at the same time-frequency locations on the spectrogram of the stimuli on which the CIm is based. If this is not the case, the resulting CIm would probably exhibit two clusters corresponding to the same acoustic cue, instead of one. If not addressed, this issue could put into question the method, as this alignment is not trivial for natural speech. Of course, we also do not know in advance the functional cues which have to be matched between the two targets. Two possible practical solutions can be considered:

1. Forcing the temporal alignment of the targets by using synthetic speech or by cross-splicing the constant parts of the stimuli. In the above example the first syllable /a/ was the same in the two targets. This is a very convenient solution as it also ensures that participants would not rely on trivial non-speech cues to perform the task, such as a delay in the beginning of the second syllable of one target compared to the other. However in some cases we do not want to manipulate the natural utterances of the targets, and we will have to go with a second option.
2. Calculating two separate “target-specific” CIm, based only on trials where one target was presented. Therefore, we ensure that for all stimuli considered the acoustic cues are at the same time-frequency locations. This is done simply by optimizing the GLM parameters on a subset of our data, the 5000 trials where *t*_0_ or *t*_1_ are presented, with the same regularization parameters as for the “overall” CIm (Figure 7). The resulting CIm $\hat{\beta }$_0_ and $\hat{\beta }$_1_ are of course noisier than previous ones, because they each rely on the half of the data, but they are helpful in checking that the position of functional cues does not differ when participants are presented with one target signal or the other. The “target-specific” CIm will be discussed in more detail in the next section. This last point raises the broader issue of non-linearities in the processing of the input stimuli.

### Non-linearity of the auditory system

Our model is derived from Equation (2) defining the decision rule for a linear observer. As for all studies involving any classification image technique, we modeled the real observer performing the identification task as a template-matcher linearly combining the input sound and a decision template to calculate a decision variable. It should be noted, however, that information processing throughout the human auditory system is obviously non-linear (Goldstein, 1967; Moore, 2002).

A first type of non-linearity already mentioned occurs when the listener's strategy is not identical when targets *t*_0_ or *t*_1_ are presented. This phenomenon can be revealed by estimating two separate CIm $\hat{\beta }$_0_ and $\hat{\beta }$_1_ based on the trials where the target signals *t*_0_ or *t*_1_ were presented, respectively (Figure 7). Differences between the two estimates for a given observer are generally interpreted as evidence for non-linearities in the auditory system, the template used for detection depending on the input signal [Abbey and Eckstein (2006)]. For all participants the critical patterns of positive and negative weights show up at the same time-frequency locations, although sometimes less clearly because they are estimated with only 5000 trials. As expected, for the ideal template-matcher case, $\hat{\beta }$_0_ and $\hat{\beta }$_1_ are very close because this simulated observer is actually implemented as a linear algorithm involving a single template. Similarly, for real listeners, differences between the estimated templates appear to be less visible than in other studies involving a discrimination signal-present vs. signal-absent task (Ahumada, 2002; Thomas and Knoblauch, 2005). Note that in our experiment the amount of phase uncertainty is reduced by the presentation of a signal in both conditions.

We additionally assumed here that non-linearities in the auditory system may be locally approximated by a linear function within the SNR range studied, a hypothesis supported by the local linearity of psychometric functions (Abbey and Eckstein, 2006). To explore this assumption empirically, we estimated the model parameters for all participants by taking into account only trials with signal contrast in the linear part of their psychometric function. The resulting CIm are very similar to those obtained previously on the whole dataset.

Furthermore, even if higher-order computations are involved, the actual mechanisms of phoneme categorization are very likely to rely on time-frequency regions highlighted by our CIm because, to some extent, noise in these regions predicts the response of the participant. In that sense, the literature on visual tasks suggests that even when the strategy used by observers is clearly non-linear, CIm may still be informative about the time-frequency location of the cues involved in the categorization mechanism. As a second step, some of these studies investigate specific non-linear effects to account for the observed divergence from linearity in their results, such as spatial or phase uncertainty (Barth et al., 1999; Murray et al., 2005; Abbey and Eckstein, 2006).

### Adaptive SNR

**Figure 8:**
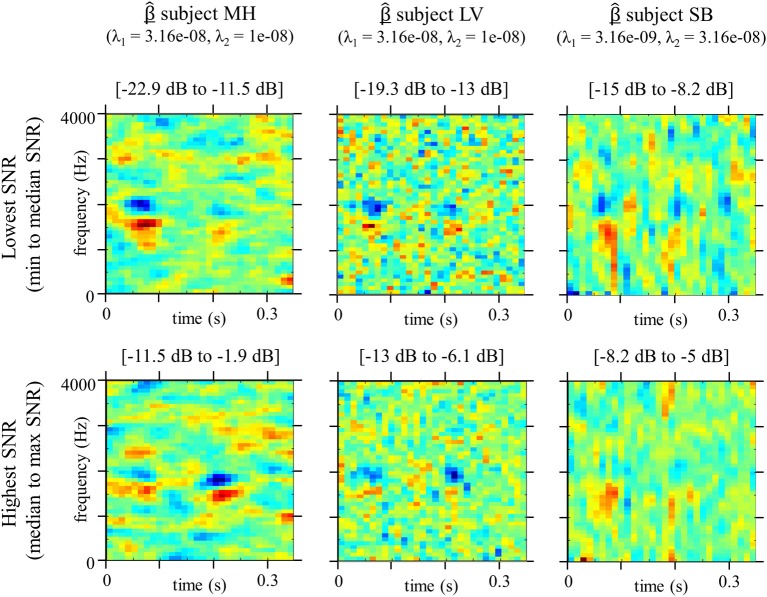
**Classification Images $\hat{\beta }$ for conditions lowest SNR (min to median SNR) or highest SNR (median to max SNR), estimated using GLM approach with smoothness priors (*n* = 5000 trials for each estimate)**. Hyperparameters values are the same as for the “overall” Classification Images Figure 4. Weights are divided by their maximum absolute values.

Another related theoretical issue of interest here relates to the use of an adaptive-SNR method. When gathering together data from the whole experiment in order to calculate a classification image, we assume that the observer's strategy for phoneme categorization in noise does not change drastically with SNR. However, this is somewhat unlikely as a number of neurophysiological studies have highlighted significant changes in cortical activity with the level of acoustic degradation of speech sounds (Obleser et al., 2008; Miettinen et al., 2010; Obleser and Kotz, 2011; Wild et al., 2012). This led us to investigate the effect of SNR on the classification image, by estimating the model parameters only on the half of the dataset with the lowest SNR (from min to median for each participant) and on the half of the dataset with the lowest SNR (from median to max) separately (Figure 8). The result seems to indicate a difference in processing within the categorization mechanism: while for low SNR the estimated templates exhibits stronger weights in absolute value on the first cue, for high SNR participants appear to rely equivalently on both cues, maybe even more on the second F2 transition. A simple explanation for this phenomenon could be that, when the noise fully masks the signal, the only remaining indicator to temporally track the relevant cues is the onset of the stimulus. Therefore, temporal uncertainty is stronger on the latest cue, resulting in more dispersed weights on the second F2 transition. This example underlines that categorization mechanisms in noise do depend on SNR level and that a lower signal contrast can bias estimated weights toward earliest cues. Further developments and studies will thus be dedicated to studying the evolution of functional fine acoustic cues with SNR value and also to adapting the methods in order to account for this influence (limiting the allowed SNR range for example).

## Conclusions

We have shown how an adaptation of a GLM with smoothness priors provides a suitable and powerful framework to investigate the way in which the human speech perception system achieves fast and efficient categorization of phonemes in noise and to estimate how human observers differ from ideal template matchers. Further developments and improvements of this method can be derived from the visual classification image literature (i.e., generalizing to multiple response alternatives and rating scales, see Dai and Micheyl (2010) and Murray et al. (2002)). Additionally, the possibility of calculating CIm in non-Gaussian noise makes it feasible to extend our method to more ecological situations as complex as speech-in-speech listening situations for example (Hoen et al., 2007; Boulenger et al., 2010); a situation that is well known to cause particular challenges in certain speech-development pathological conditions, for example dyslexia (Ziegler et al., 2009; Dole et al., 2012). Further developments should also deal with the issues of realizing and analyzing group studies.

### Conflict of interest statement

The authors declare that the research was conducted in the absence of any commercial or financial relationships that could be construed as a potential conflict of interest.
